# The prognostic significance of VISTA and CD33-positive myeloid cells in cutaneous melanoma and their relationship with PD-1 expression

**DOI:** 10.1038/s41598-020-71216-2

**Published:** 2020-09-01

**Authors:** Jae Won Choi, Young Jae Kim, Kyung A. Yun, Chong Hyun Won, Mi Woo Lee, Jee Ho Choi, Sung Eun Chang, Woo Jin Lee

**Affiliations:** Department of Dermatology, Asan Medical Center, University of Ulsan College of Medicine, 88, Olympic-ro 43-gil, Songpa-gu, Seoul, 05505 Korea

**Keywords:** Cancer, Immunology, Medical research, Oncology

## Abstract

V-domain Ig suppressor of T-cell activation (VISTA), which mediates immune evasion in cancer, is mainly expressed on hematopoietic cells and myeloid cells in the tumor. We evaluated correlations among the expression of VISTA, the myeloid-derived suppressor cell marker CD33, and programmed death-1 (PD-1), and determined their relationships with clinicopathological characteristics and disease outcomes in melanoma. Diagnostic tissue from 136 cases of melanoma was evaluated by immunohistochemistry for CD33, VISTA, and PD-1 expression. Dual immunofluorescence using CD33 and VISTA antibodies was performed. VISTA expression positively correlated with CD33 expression in melanoma tissue. Dual immunofluorescence staining revealed that VISTA was expressed by CD33-positive myeloid cells. PD-1 expression correlated with CD33 and VISTA expression. CD33 and VISTA expression were significantly associated with negative prognostic factors, including a deeper Breslow thickness and an advanced stage of disease. High expression of either CD33 or VISTA was associated with worse survival. Positivity for both VISTA and PD-1 predicted worse survival. Multivariate analysis showed that both CD33 and VISTA expression were independent prognostic factors in cutaneous melanoma. VISTA and CD33 expression are independent unfavourable prognostic factors in melanoma, which suggests their potential as therapeutic targets.

## Introduction

Myeloid-derived suppressor cells (MDSCs) are bone marrow-derived myeloid progenitors that include macrophages, granulocytes, dendritic cells, and immature myeloid cells^1,2^. MDSCs play important roles in disease progression and immune evasion in many types of cancer^3–5^. There are two subpopulations of MDSCs, monocytic MDSCs and granulocytic MDSCs^6,7^. MDSCs typically express the common myeloid markers CD33 and CD11b^8^.

V-domain Ig suppressor of T-cell activation (VISTA) is a recently identified negative regulator of T-cell-mediated immune responses in cancer^9–12^. In contrast to programmed cell death protein-1 (PD-1), which is primarily found on activated and exhausted T cells, VISTA is mainly expressed on hematopoietic cells and myeloid cells including neutrophils, monocytes, macrophages, and dendritic cells^13,14^. Expression of VISTA is high in myeloid cells and low in CD4+ and CD8+ T cells and CD4+ FoxP3+ regulatory T cells^9–11,15^. As an immune checkpoint inhibitor with a different expression pattern than other previously identified checkpoints, VISTA could be a novel therapeutic marker in anticancer immunotherapy^10^. VISTA overexpression downregulates the immunity by suppression T-cell proliferation and production of T-cell cytokine such as IL-2 and IFN-γ^11^. The inhibitory function of VISTA in anticancer immunity was demonstrated in mice transplanted with melanoma, in which blocking of VISTA induced antitumor immunity by increasing tumor-specific CD4+ and CD8+ T cells and decreasing FoxP3+ regulatory T cells in the tumor microenvironment.10 Genetic deletion of VISTA (*Vsir*) resulted in increased production of inflammatory cytokines and chemokines in a mice model^16^.

Recent study noticed the role of MDSC-mediated immunosuppression in tumor progression and found that MDSCs can contribute to patient resistance to immune checkpoint inhibition^17^. It was also reported that reversed MDSC-mediated suppression with myeloid cell receptor tyrosine kinases inhibition consequences the augmentation of anti-PD-1 therapy in melanoma^18^.

These findings imply close underlying correlation between MDSCs and PD-1 in tumor microenvironment. Likewise, the expression of VISTA positively correlated with the expression of PD-ligand 1 (PD-L1) and PD-1, and the numbers of CD8+ T cells and CD68+ macrophages, in the tumor microenvironment in non-small cell lung cancer^19^. In oral squamous cell carcinoma, VISTA expression correlated with MDSC markers (CD11b and CD33) and was associated with increased expression of IL13Rα2, an important cytokine involved in tumor metastasis and recruitment of MDSCs^20^.

Although the prognostic significance of VISTA and its correlation with PD-1 expression has been evaluated in cutaneous melanoma^21^, the association between VISTA and CD33+ MDSCs has not been elucidated. Recently, combinations of immune checkpoint inhibitors have emerged as anticancer therapies. For example, CA-170, is an oral inhibitor which targets both PD-L1 and VISTA revealed remarkable anti-tumor effects in preclinical study and the phase I trial in patients with advanced solid tumors including melanoma is currently investigated (NCT02812875)^22^. In this aspect, evaluation of the relationship between PD-1 and VISTA or CD33 expression is important. However, many questions are still remained for VISTA and its interaction with other immune check point molecules.

In the present study, we examined the expression of VISTA in primary melanoma tissue and analyzed its association with clinicopathological features and clinical outcome. In addition, the correlation between the expression of VISTA and MDSC markers was evaluated. We also analyzed the association between CD33/VISTA and PD-1 to evaluate the potential for combination therapy with MDSC-targeting agents and anti-PD-1 antibodies.

## Materials and methods

This study was approved by the Institutional Review Board of Asan Medical Center. A database at Asan Medical Center was searched for cases of cutaneous melanoma that were confirmed by skin biopsy between January 2001 and December 2016. All experiments were performed in accordance with relevant guidelines and regulations. Informed consent to publish identifying informations was obtained from study participants.

### Evaluation of VISTA and CD33 expression in cutaneous melanoma

Paraffin-embedded sections were immunostained with anti-VISTA (1:200, ProSci Incorporation, CA, USA), anti-CD33 (1:100, Leica Biosystems, Newcastle, UK), or anti-PD-1 (1:100, Ventana, Tucson, AZ, USA) antibodies. All slides were evaluated and scored under light microscopy, analyzed and photographed by using Olympus cellSens software (version 1.4). Tumor-infiltrating inflammatory cells (TIICs) were identified based on morphology in hematoxylin–eosin (H&E)-stained sections. Immunohistochemical staining was measured by semiquantitative assessment of positive staining in TIICs. For VISTA, CD33, and PD-1 antibodies, the percentage of TIICs showing positive membrane staining was determined. The intensity of staining was determined on a scale of 0–3 (0 =  < 5%, 1 = 5–20%, 2 = 20–50%, and 3 =  > 50% of TIICs). Cases with a score ≥ 1 were considered positive. Scores of 2 or 3 were considered to represent high expression of VISTA, CD33, and PD-1. The numbers of VISTA-, PD-1-, and CD33-positive TIICs were counted in five representative high-powered fields (200X magnification) and averaged. All melanoma tissues evaluated in the study are the primary tumor tissues from the primary cutaneous melanoma tissues. All samples were evaluated independently by two investigators (LWJ, CJW).

### Immunofluorescence staining

Dual immunofluorescence (IF) staining was carried out using formalin-fixed, paraffin-embedded sections after heat antigen retrieval following standard protocols. The following primary antibodies were used: anti-VISTA (1:50, ProSci Incorporation, Poway, CA, USA), anti-CD33 (1:50, Leica Biosystems, Newcastle, UK), and anti-PD-1 (1:50, R&D Systems, Minneapolis, MN, USA). The secondary antibodies and dilutions used for IF staining were as follows: goat anti-rabbit IgG(H+L)-FITC (Southern Biotech, Birmingham, AL, USA; cat. no. 4055-02; 1:500), rabbit anti-goat IgG(H+L)-FITC (Southern Biotech; cat. no. 6160-02, 1:500), and cross-adsorbed goat anti-mouse IgG(H+L)-AlexaFluor 546 (ThermoFisher Scientific, Waltham, MA, USA; cat. no. A-11003; 1:500).

### Variables of interest

The clinical features of the patient and the primary lesion, including age at diagnosis, sex, the location of the lesion, lymph node (LN) invasion, and distant visceral metastasis, were determined through the medical records and preoperative photographs. Overall survival (OS) was calculated from the date of the initial diagnosis to either the date of death from any cause or the date of the last follow-up examination. Progression-free survival (PFS) was calculated from the date of the initial diagnosis to the first day of disease progression or recurrence, or the last follow-up. Survival curves were calculated from medical records. The stage of disease was determined according to the 8th edition of the American Joint Committee on Cancer (AJCC)^23^. Biopsy specimens were reviewed and the following data was collected: histopathologic subtype, Breslow thickness, ulceration, and vertical growth phase.

### Statistical analysis

All analyses were performed using a statistical software package (SPSS, version 18.0; SPSS Inc., Chicago, IL). P-values of less than 0.05 were considered statistically significant. Survival was analyzed using the Kaplan–Meier method, and comparisons were made by log-rank testing. Prognostic factors independently associated with survival were identified by multivariate analysis using Cox proportional hazards regression modelling. Comparisons between subgroups were performed using a Chi-squared test or Fisher’s exact test for categorical variables, and a t-test or the Mann–Whitney test for continuous variables. Pearson’s correlation coefficient was used to evaluate associations among continuous variables.

## Results

A total of 136 cases of cutaneous melanoma were included in the study. The demographic data and clinical features of the patients are summarized in Table 1.

**Table 1:** Clinico-histopathological characteristics of the 136 cutaneous melanomas according to CD33, VISTA and PD-1 expression.

	CD33 expression			VISTA expression			PD-1 expression		
	Score 0 or 1 (*n* = 93)	Score 2 or 3 (*n* = 43)	*P *value	Score 0 or 1 (*n* = 99)	Score 2 or 3 (*n* = 37)	*P*-value	Score 0 or 1 (n = 69)	Score 2 or 3 (n = 67)	*P *value
**Breslow thickness, mm**			0.009*			0.002*			0.088
≤ 1 (T1) (*n* = 24)	20/93 (21.5)	4/43 (9.3)		20/99 (20.2)	4/37 (10.8)		15/69 (21.7)	9/67 (13.4)	
> 1 to ≤ 2 (T2) (*n* = 49)	37/93 (39.8)	12/43 (27.9)		41/99 (41.4)	8/37 (21.6)		27/69 (39.1)	22/67 (32.8)	
> 2 to ≤ 4 (T3) (*n* = 35)	25/93 (26.9)	10/43 (23.3)		25/99 (25.3)	10/37 (27.0)		19/69 (27.5)	16/67 (23.9)	
> 4 (T4) (*n* = 28)	11/93 (11.8)	17/43 (39.5)		13/99 (13.1)	15/37 (40.5)		8/69 (11.6)	20/67 (29.9)	
**Ulceration**			0.040*			0.015*			0.210
Yes (*n* = 38)	21/93 (22.6)	17/43 (39.5)		22/99 (22.2)			16/69 (23.2)	22/67 (32.8)	
No (*n* = 98)	72/93 (77.4)	26/43 (60.5)		77/99 (77.8)	16/37 (43.2)		53/69 (76.8)	45/67 (67.2)	
**Vertical growth phase**			0.063		21/37 (56.8)	0.041*			0.452
Yes (*n* = 51)	30/93 (32.3)	21/43 (48.8)		32/99 (32.3)	19/37 (51.4)		28/69 (40.6)	23/67 (34.3)	
No (*n* = 85)	63/93 (67.7)	22/43 (51.2)		67/99 (67.7)	18/37 (48.6)		41/69 (59.4)	44/67 (65.7)	
**Sex**									
Male (*n* = 74)	46/93 (49.5)	28/43 (65.1)	0.088	55/99 (55.6)	19/37 (51.4)	0.661	39/69 (56.5)	35/67 (52.7)	0.616
Female (*n* = 62)	47/93 (50.5)	15/43 (34.9)		44/99 (44.4)	18/37(48.6)		30/69 (43.5)	32/67 (47.8)	
**Age**									
< 60 years (*n* = 74)	54/93 (58.1)	20/43 (46.5)	0.208	56/99 (56.6)	18/37 (48.6)	0.409	36/69 (52.2)	38/67 (56.7)	0.595
≥ 60 years (*n* = 62)	39/93 (41.9)	23/43 (53.5)		43/99 (43.4)	19/37 (51.4)		33/69 (47.8)	29/67 (43.3)	
**Extracutaneous involvement**									
Lymph node (*n* = 28)	12/93 (12.9)	16/43 (37.2)	0.001*	13/99 (13.1)	15/37 (40.5)	0.000*	17/69 (24.6)	11/67 (16.4)	0.236
Viscera (*n* = 12)	5/93 (5.4)	7/43 (16.3)	0.051	6/99 (6.1)		0.063	6/69 (8.7)	6/67 (9.0)	0.957
**AJCC stage**			0.005*		6/37 (16.2)	0.008*			0.192
I/II (*n* = 103)	77/93 (82.8)	26/43 (60.5)		81/99 (81.8)	22/37 (59.5)		49/69 (71.0)	54/67 (80.6)	
III/IV (*n* = 33)	16/93 (17.2)	17/43 (39.5)		18/99 (18.2)	15/37 (40.5)		20/69 (29.0)	13/67 (19.4)	

*Statistically significant.

VISTA, V-domain Ig suppressor of T-cell activation; AJCC, American Joint Committee on Cancer.

### Expression of CD33 and VISTA and their association with clinicopathological features

**Figure 1:**
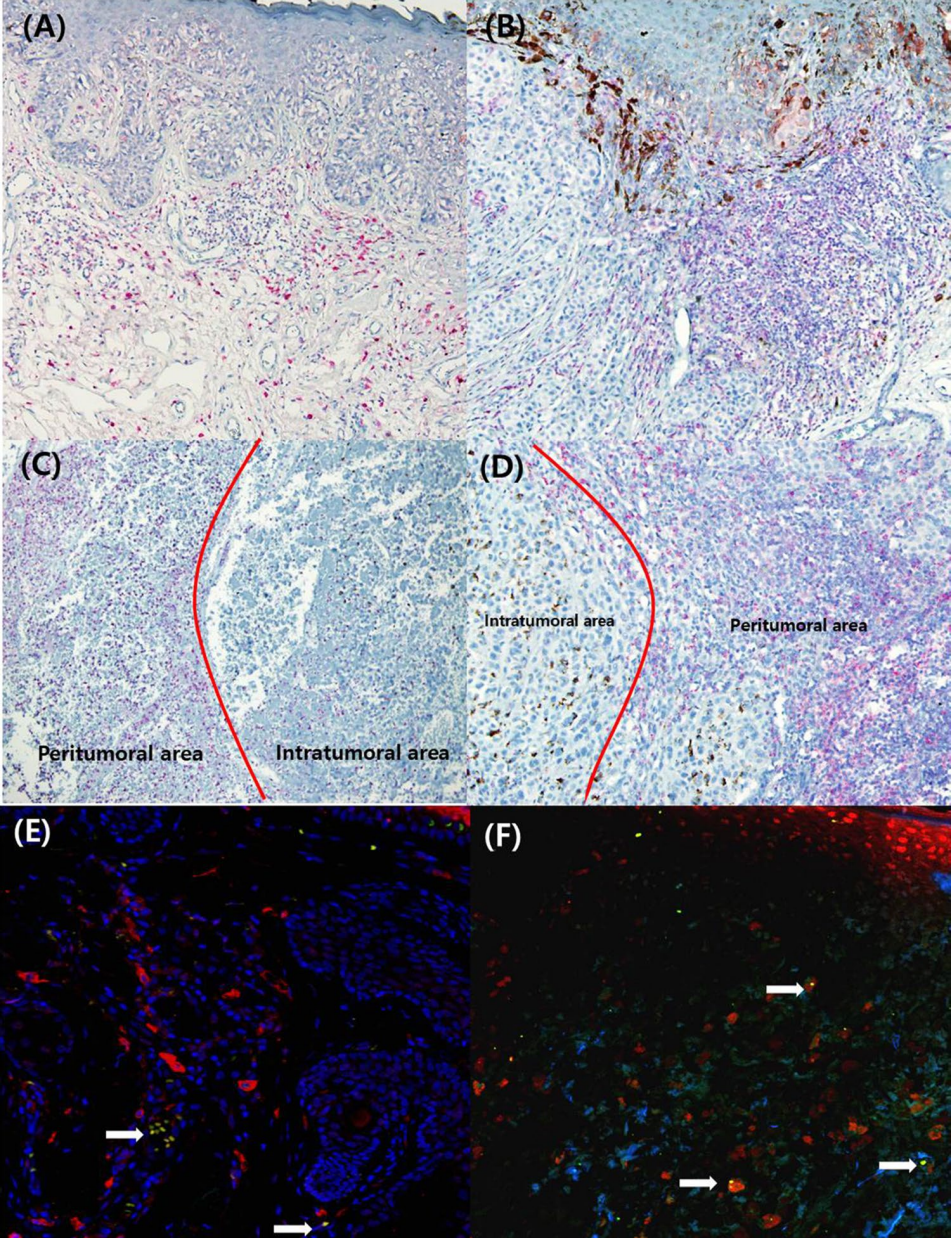
Expression of CD33 and VISTA in cutaneous melanoma. (**A**) High intratumoral expression (immunohistochemical score of 3, red color, cytoplasmic) of CD33 in acral melanoma (× 100). (**B**) High intratumoral expression (score 3, red color, cytoplasmic) of VISTA in nodular melanoma (× 100). (**C**) High peritumoral expression (score 3, red color, cytoplasmic) of CD33 in acral melanoma (× 100). (**D**) High peritumoral expression (score 3, red color, cytoplasmic) of VISTA in nodular melanoma (× 200). (**E**) Dual immunofluorescence staining of (**A**) CD33 and VISTA (CD33, red, cytoplasmic; VISTA, green, cytoplasmic) and (**F**) PD-1 and VISTA (PD-1, red, cytoplasmic; VISTA, green, cytoplasmic). Double-positive cells are shown in yellow (white arrow). VISTA, V-domain Ig suppressor of T-cell activation; PD-1, programmed cell death protein-1.

Of 136 patients, CD33 (Fig. 1A,C) and VISTA (Fig. 1B,D) were highly expressed in 43 (31.6%) and 37 (27.2%) patients, respectively. Clinicopathological variables were stratified by the expression of these proteins in the tumor to identify correlations (Table 1).

High expression of CD33 was associated with clinicopathological variables that have negative prognostic indexes. There were significant correlations between high expression of CD33 and pathological variables such as a deeper Breslow thickness (*P* = 0.009) and the presence of ulceration (*P* = 0.040). High CD33 expression was also associated with a higher likelihood of LN involvement (*P* = 0.001) and an advanced AJCC stage (*P* = 0.005; Table 1).

High VISTA expression was also associated with poor clinicopathological features. There was a significant association between high expression of VISTA and pathological features such as a deeper Breslow thickness (*P* = 0.002), the presence of ulceration (*P* = 0.015) and higher frequency of vertical growth phase (*P* = 0.041). More tendency of LN involvement (*P* < 0.001) and advanced AJCC stage (*P* = 0.008) (Table 1) were also associated with high expression of VISTA.

Of 136 patients, 67 (49.3%) showed high PD-1 expression (immunohistochemical score of 2 or 3). However, high PD-1 expression was not associated with clinicopathological variables that have negative prognostic indexes, such as a deeper Breslow thickness (*P* = 0.088), the presence of ulceration (*P* = 0.210) and higher frequency of vertical growth phase (*P* = 0.452). Among these 67 patients, 27 (40.3%) and 24 (35.8%) showed high expression of CD33 and VISTA, respectively (Table 2). There was a significant correlation between PD-1 expression and expression of CD33 (*P* = 0.032) and VISTA (*P* = 0.026).

**Table 2:** Correlations among CD33, VISTA, and PD-1 expression.

	VISTA expression, n (%)		*P *value	CD33 expression, n (%)		*P *value
	Score 0 or 1 (*n* = 99)	Score 2 or 3 (*n* = 37)		Score 0 or 1 (*n* = 93)	Score 2 or 3 (*n* = 43)	
**CD33 expression**			< 0.001*			
Score 0 or 1 (*n* = 93)	88/99 (88.9)	5/37 (13.5)		NA		
Score 2 or 3 (*n* = 43)	11/99 (11.1)	32/37 (86.5)				
**PD-1 expression**			0.026*			0.032*
Score 0 or 1 (*n* = 69)	56/99 (56.6)	13/37 (35.1)		53/93 (57.0)	16/43 (37.2)	
Score 2 or 3 (*n* = 67)	43/99 (43.4)	24/37 (64.9)		40/93 (43.0)	27/43 (62.8)	

*Statistically significant.

VISTA, V-domain Ig suppressor of T-cell activation; PD-1, programmed cell death protein-1.

### Expression of VISTA on CD33-positive MDSCs

Double IF staining of melanoma tissue showed that CD33-positive cells also expressed VISTA (Fig. 1E). Of 43 cases with high CD33 expression, 32 (74.4%) also showed high expression of VISTA. There was a significant correlation between the expression of CD33 and the expression of VISTA (Table 2, *P* < 0.001). Double IF staining showed that PD-1-positive cells also expressed VISTA (Fig. 1F).

### Prognostic significance of CD33 and VISTA expression

When all patients were combined into a single cohort, the 5-year OS rate was 52%, and the median OS was 62.0 months [95% confidence interval (CI) 50.12–79.52 months].

**Figure 2:**
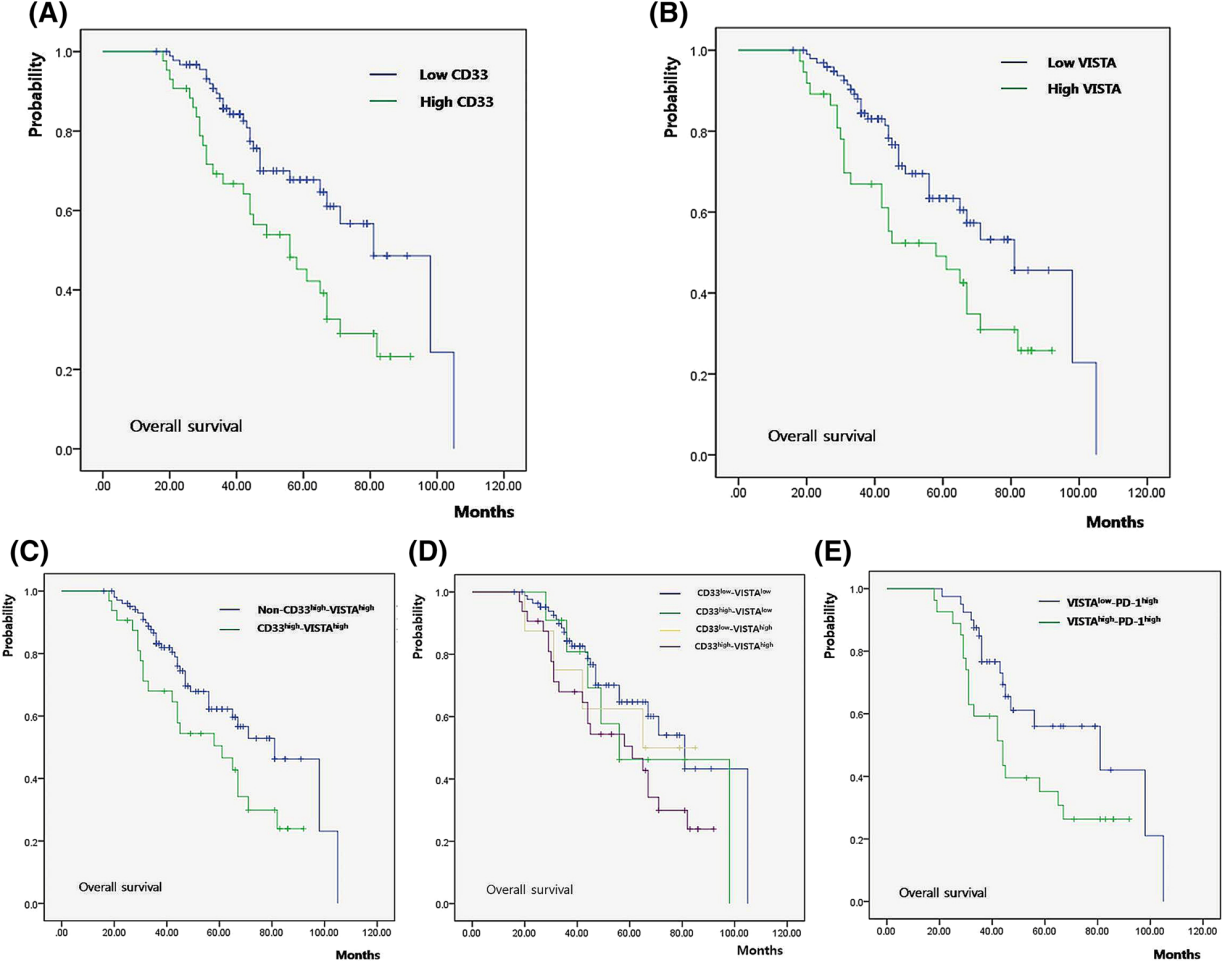
Patient survival according to CD33 and VISTA expression. (**A**) OS according to CD33 expression. (**B**) OS according to VISTA expression. (**C**) Worse OS in patients with high expression of both CD33 and VISTA. (**D**) OS according to the degree of CD33 and VISTA expression (**E**) Worse OS in cases with dual expression of VISTA and PD-1 among patients with high expression of PD-1. VISTA, V-domain Ig suppressor of T-cell activation; PD-1, programmed cell death protein-1.

Median OS was significantly shorter in patients with high expression of CD33 (56.0 months; 95% CI 38.51–73.49 months) compared with those with low expression of CD33 (81.0 months; 95% CI 64.64–97.35 months) (*P* = 0.004, Fig. 2A). PFS was also significantly better in patients with low expression of CD33 (*P* < 0.001). Patients with high expression of VISTA (58.0 months; 95% CI 30.55–85.47 months) had inferior median OS compared with patients with low expression of VISTA (79.0 months; 95% CI 62.00–95.42 months) (*P* = 0.017, Fig. 2B). PFS was also affected by the expression of VISTA (*P* = 0.008). High expression of both CD33 and VISTA predicted inferior median OS (CD33^high^VISTA^high^: 61.0 months, 95% CI 35.65–86.35 months; and non-CD33^high^VISTA^high^: 79.0 months, 95% CI 63.09–94.87 months) (*P* = 0.032, Fig. 2C) and PFS (*P* = 0.004). Patients with low expression of both CD33 and VISTA (CD33^low^VISTA^low^: 81.0 months, 95% CI 61.39–199.60 months) showed significantly better median OS than patients with high expression of either CD33 or VISTA (Fig. 2D, *P* = 0.027, CD33^high^VISTA^low^: 56.0 months, 95% CI 28.28–83.72 months; and CD33^low^VISTA^high^: 65.0 months, 95% CI not found). When survival was evaluated with respect to PD-1 expression, high expression of both VISTA and PD-1 was associated with worse median OS (VISTA^high^PD-1^high^: 44.0 months, 95% CI 30.4–57.59 months; and non-VISTA^high^PD-1^high^: 71.0 months, 95% CI 59.49–82.50 months) (*P* = 0.033). High expression of VISTA was also associated with OS in patients with high expression of PD-1 (VISTA^high^PD-1^high^: 44.0 months, 95% CI 30.78–60.21 months; and VISTA^low^PD-1^high^: 81.0 months, 95% CI 31.82–111.17 months) (*P* = 0.036, Fig. 2E).

By univariate analysis, CD33 expression [hazard ratio (HR) 2.11; 95% CI 1.21–4.23; *P* = 0.031] and VISTA expression (HR 1.86; 95% CI 1.26–5.54; *P* = 0.039) were associated with worse OS (Table 3). By multivariate analysis using these variables, CD33 expression (HR 1.61; 95% CI 1.18–4.23; *P* = 0.041) and VISTA expression (HR 1.51; 95% CI 1.14–5.61; *P* = 0.046) were independent prognostic markers for worse OS (Table 3).

**Table 3:** Univariate and multivariate analyses of OS and PFS.

Covariate	OS			PFS		
	HR	95% CI	*P *value	HR	95% CI	*P *value
**Univariate analysis**						
**AJCC stage**						
Early versus advanced	2.41	1.24–4.28	0.023*	2.19	1.14–5.37	0.045*
**Breslow thickness**						
T1 or T2 versus T3 or T4	2.19	1.21–5.55	0.028*	1.98	1.29–6.11	0.039*
**CD33 expression**						
High versus low	2.11	1.21–4.23	0.031*	1.98	1.16–4.55	0.042*
**VISTA expression**						
High versus low	1.86	1.26–5.54	0.039*	1.43	1.05–4.77	0.053
**PD-1 expression**						
High versus low	0.745	0.31–3.29	0.573	0.642	0.33–3.12	0.528
**Multivariate analysis**						
**AJCC stage**						
Early versus advanced	1.89	1.21–4.59	0.042*	1.87	1.11–4.21	0.051
**Breslow thickness**						
T1 or T2 versus T3 or T4	1.84	1.21–4.33	0.044*	1.35	1.02–4.77	0.064
**CD33 expression**						
High versus low	1.61	1.18–4.23	0.041*	1.66	1.09–4.98	0.056
**VISTA expression**						
High versus low	1.51	1.14–5.61	0.046*	1.21	1.04–5.11	0.040*

*Statistically significant.

VISTA, V-domain Ig suppressor of T-cell activation; PD-1, programmed cell death protein-1; AJCC, American Joint Committee on Cancer; OS, Overall survival; PFS, progression-free survival; CI, confidence interval; HR, hazard ratio.

## Discussion

Immune checkpoints and suppressive immune cells such as MDSCs, regulatory T cells, and tumor-associated macrophages play important roles in tumor progression by enabling cancer cells to evade antitumor immunity^24,25^. MDSCs are a heterogeneous group of myeloid cells that suppress T-cell activity in the tumor microenvironment^26^. MDSCs can contribute to tumor invasion by inhibiting T-cell proliferation as well as by promoting tumor metastasis and angiogenesis^26–30^. Activated MDSCs in tumors contribute to the immunosuppressive microenvironment by producing arginine, reactive oxygen species, and immunosuppressive cytokines^31,32^. In addition, MDSCs express PD-L1 in response to tumor hypoxia and tumor-derived exosomes^33,34^. PD-1 expression in tumor-associated MDSCs is related to their proliferation and expression of molecules that inhibit the activity of antitumor T cells^35^. Therefore, blocking MDSCs could be a novel anticancer strategy that could enhance the antitumor effects of PD-1/PD-L1 inhibitors.

CD33 is a transmembrane receptor and myeloid differentiation antigen that is expressed on myeloid cells and is over-expressed in 90% of myeloid leukemias^36^. CD33 is found on maturing granulocytes, monocytes, and multipotent myeloid precursors, and is also expressed on subsets of activated T cells, natural killer cells, and B cells^37–39^. Because of this expression pattern, CD33-directed therapies are used in malignancies such as chronic myelomonocytic leukemia, myelodysplastic syndrome, and acute lymphoblastic leukemia^40–42^.

The expression of CD33+ MDSCs in cutaneous melanoma and its relationship with PD-1 expression has not been evaluated. In this study, about 30% of melanoma specimens showed high expression of CD33. Furthermore, high expression of CD33 was associated with poor clinicopathological variables and was an independent prognostic factor in melanoma. Moreover, CD33 expression correlated with PD-1 expression, which suggests the possibility that PD-1 expression could be affected by CD33-positive MDSCs in cutaneous melanoma.

As a negative immune checkpoint molecule with a different expression pattern than other previously described immune checkpoints, VISTA could be a novel therapeutic marker in anticancer immunotherapy^10^. VISTA overexpression downregulates the immunity by suppression T-cell proliferation and production of T-cell cytokine such as IL-2 and IFN-γ^11^. The inhibitory function of VISTA in anticancer immunity was demonstrated in mice transplanted with melanoma, in which blocking of VISTA induced antitumor immunity by increasing tumor-specific CD4+ and CD8+ T cells and decreasing FoxP3+ regulatory T cells in the tumor microenvironment^10^. Genetic deletion of VISTA (*Vsir*) resulted in increased production of inflammatory cytokines and chemokines in a mice model^16^.

Consistent with previous data that VISTA is mainly expressed on hematopoietic cells and myeloid cells, and not exhausted or activated T cells, our study showed that VISTA expression correlated with the expression of the MDSC marker CD33 in cutaneous melanoma. Thirty-two of forty-three (74.4%) patients with high expression of CD33 had high expression of VISTA. Double IF showed that CD33-positive MDSCs expressed VISTA. In a recent review of VISTA expression, high VISTA expression was found in myeloid cells, specifically microglia and neutrophils followed by monocytes, macrophages, and dendritic cells. Regarding T lymphocytes, VISTA is most highly expressed on naïve CD4+ and FoxP3+ regulatory T cells^43^.

VISTA blockade in the B16 melanoma model reduced the number of tumor-infiltrating monocytic MDSCs and increased the density of tumor-infiltrating effector T cells^10^. VISTA expression positively correlated with the expression of PD-1, PD-L1, and cytotoxic T-lymphocyte–associated antigen 4 (CTLA-4) in tumor-infiltrating immune cells, suggesting that these molecules simultaneously contribute to evasion of antitumor immunity and play a role in cancer progression^20,21^. We also found that VISTA expression was associated with clinicopathologic variables that are indexes of disease progression and poor survival, suggesting that VISTA is a prognostic biomarker and could be a target of antitumor immunotherapy. Furthermore, patients with high expression of both CD33 and VISTA had the worst survival in our cohort. Expression of VISTA on CD33-positive MDSCs could enhance their immunosuppressive function, resulting in tumor progression. In the present study, VISTA expression also correlated with PD-1 expression in melanoma tissue. The frequency of high expression was higher in PD-1 than VISTA in presenting study. We found that VISTA and PD-1 were both highly expressed in approximately 30% of melanoma cases. Among patients with high expression of PD-1, patients with high expression of VISTA (VISTA^high^PD-1^high^) had a worse prognosis than patients with low expression of VISTA (VISTA^low^PD-1^high^). These data suggest that VISTA expression can stratify survival in patients with high expression of PD-1. This finding also suggests that combination therapy with anti-VISTA and anti-PD-1 antibodies may be an effective approach for immunotherapy of cutaneous melanoma.

The combination of VISTA and PD-1 inhibitors synergistically enhanced antitumor immune responses in mouse models^10,44^. This suggests the therapeutic potential of targeting VISTA as an anticancer strategy. VISTA expression was previously reported to be an independent prognostic factor in melanoma^21^. Adaptive resistance to anti-PD-1 therapy also correlated with VISTA upregulation in melanoma patients, suggesting that VISTA blockade, possibly combined with PD-1 blockade, represents a potentially efficacious treatment strategy^45^. In addition, combination blockade of PD-1 and VISTA in a mice model did not result in overt autoimmunity, suggesting that this regimen could represent a less toxic alternative to anti-PD-1 and anti-CTLA-4 combination regimens^44^.

In conclusion, our study suggests that studies on the expression of CD33 and VISTA in melanoma may be of pivotal future interest. In light of the co-expression of PD-1 and VISTA, VISTA inhibitors may be of particular use in combination with immune checkpoint inhibitors such as PD-1 antibodies.

## Supplementary information

Supplementary Figure 1.
