# Supplementary material for: The prognostic significance of VISTA and CD33-positive myeloid cells in cutaneous melanoma and their relationship with PD-1 expression
Source: Sci Rep. 2020 Sep 1;10:14372. doi: 10.1038/s41598-020-71216-2 (PMC7462859; doi:10.1038/s41598-020-71216-2)
Supplement: Supplementary file 1 — Supplementary Figure 1. [file 41598_2020_71216_MOESM1_ESM.docx]

**The Prognostic Significance of VISTA and CD33-Positive Myeloid Cells in Cutaneous Melanoma and Their Relationship with PD-1 Expression**

Jae Won Choi^†^, Young Jae Kim^†^, Kyung A Yun, Chong Hyun Won, Mi Woo Lee, Jee Ho Choi, Sung Eun Chang, Woo Jin Lee*

Department of Dermatology, Asan Medical Center, University of Ulsan College of Medicine, 88, Olympic-ro 43-gil, Songpa-gu, Seoul 05505, Korea

^†^These authors contributed equally to this work.

*Corresponding author: Woo Jin Lee, email: uucm79@gmail.com


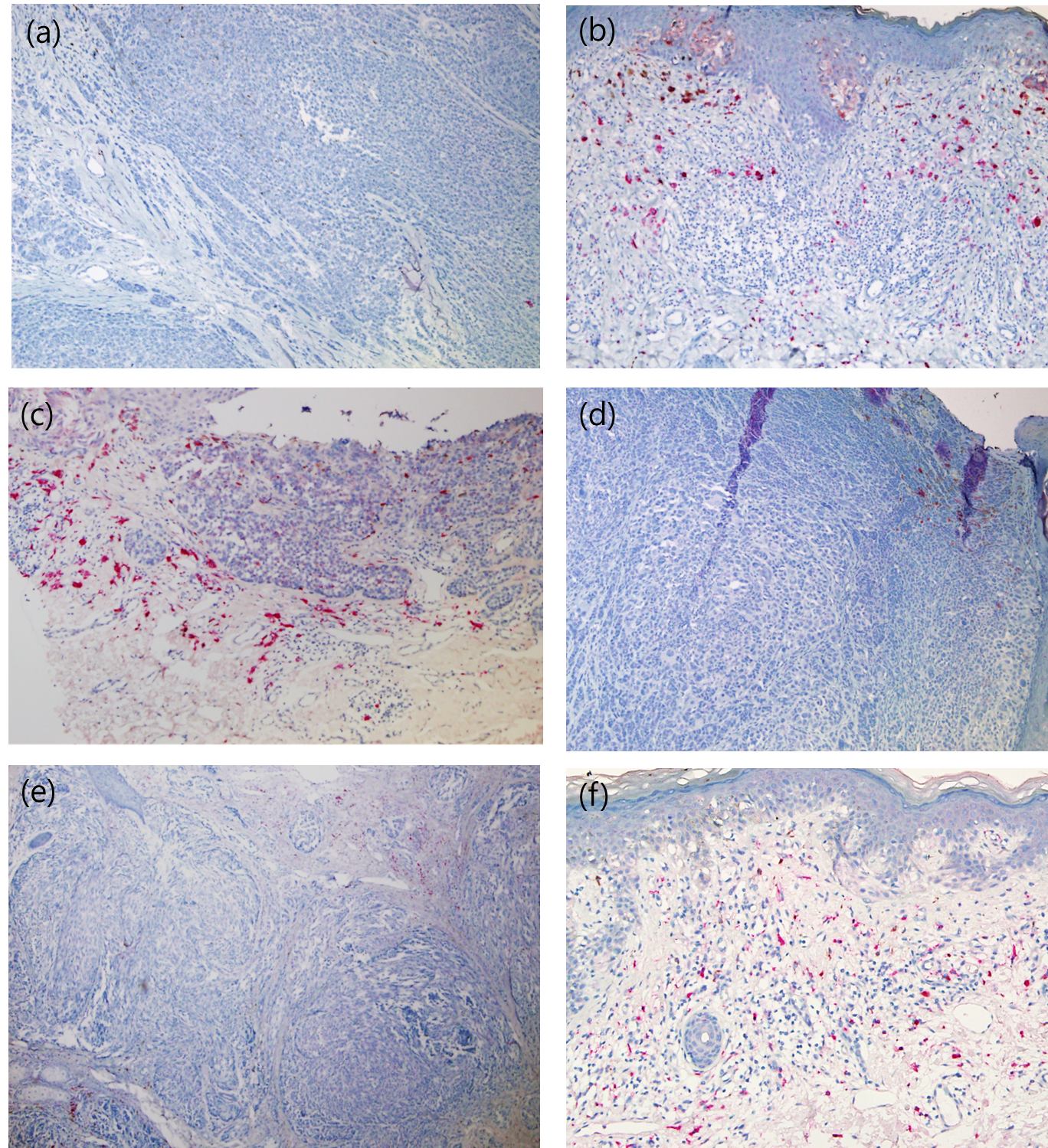


**Supplementary Figure S1.** Expression of CD33 and VISTA in cutaneous melanoma. (a) Negative intratumoral expression (immunohistochemical score of 0) of CD33 in melanoma (x100). (b) Low intratumoral expression (score 1, red color, cytoplasmic) of CD33 in melanoma (x100). (c) High peritumoral expression (score 2, red color, cytoplasmic) of CD33 in melanoma (x100). (d) Negative intratumoral expression (immunohistochemical score of 0) of VISTA in melanoma (x100). (e) Low intratumoral expression (score 1, red color, cytoplasmic) of VISTA in melanoma (x100). (f) High peritumoral expression (score 2, red color, cytoplasmic) of VISTA in melanoma (x200).
